# DEPTH2: an mRNA-based algorithm to evaluate intratumor heterogeneity without reference to normal controls

**DOI:** 10.1186/s12967-022-03355-1

**Published:** 2022-04-01

**Authors:** Dandan Song, Xiaosheng Wang

**Affiliations:** 1 Biomedical Informatics Research Lab, School of Basic Medicine and Clinical Pharmacy, China Pharmaceutical University, Nanjing, 211198 China; 2 Cancer Genomics Research Center, School of Basic Medicine and Clinical Pharmacy, China Pharmaceutical University, Nanjing, 211198 China; 3 Big Data Research Institute, China Pharmaceutical University, Nanjing, 211198 China

**Keywords:** Algorithm, Intratumor heterogeneity, Gene expression profiles, Cancer prognosis, Antitumor immunity, Genomic instability

## Abstract

**Background:**

Intratumor heterogeneity (ITH) is associated with tumor progression, unfavorable prognosis, immunosuppression, genomic instability, and therapeutic resistance. Thus, evaluation of ITH levels is valuable in cancer diagnosis and treatment.

**Methods:**

We proposed a new mRNA-based ITH evaluation algorithm (DEPTH2) without reference to normal controls. DEPTH2 evaluates ITH levels based on the standard deviations of absolute z-scored transcriptome levels in tumors, reflecting the asynchronous level of transcriptome alterations relative to the central tendency in a tumor.

**Results:**

By analyzing 33 TCGA cancer types, we demonstrated that DEPTH2 ITH was effective in measuring ITH for its significant associations with tumor progression, unfavorable prognosis, genomic instability, reduced antitumor immunity and immunotherapy response, and altered drug response in diverse cancers. Compared to other five ITH evaluation algorithms (MATH, PhyloWGS, ABSOLUTE, DEPTH, and tITH), DEPTH2 ITH showed a stronger association with unfavorable clinical outcomes, and in characterizing other properties of ITH, such as its associations with genomic instability and antitumor immunosuppression, DEPTH2 also displayed competitive performance.

**Conclusions:**

DEPTH2 is expected to have a wider spectrum of applications in evaluating ITH in comparison to other algorithms.

**Supplementary Information:**

The online version contains supplementary material available at 10.1186/s12967-022-03355-1.

## Background

Intratumor heterogeneity (ITH) refers to the differences of molecular and phenotypic profiles between different tumor cells within tumors. ITH has significant associations with tumor advancement, prognosis, immune evasion, and therapeutic responses [1]. Many algorithms have been proposed to quantify ITH at the genetic level, such as MATH [2], EXPANDS [3], DITHER [4], and PhyloWGS [5]. However, although genetic ITH is prevalent in human cancers, it cannot delineate the full phenotypic diversity of cancers [6]. It suggests that mRNA ITH could also contribute to the phenotypic diversity of cancers. Thus, a few algorithms have been developed to quantify ITH at the mRNA level, such as DEPTH [6] and tITH [7]. Previously, we developed an mRNA-based algorithm, namely DEPTH [6], to evaluate ITH levels. We demonstrated that DEPTH is superior to or comparable with other methods in characterizing the properties of ITH [6].

In this study, we proposed a novel algorithm to score ITH at the mRNA level. Similar to DEPTH, the novel algorithm, termed DEPTH2, measured ITH based on the perturbations of gene expression profiles. However, different from DEPTH and other ITH evaluation methods, DEPTH2 quantified ITH without reference to normal controls. It indicates that DEPTH2 can be applied to any gene expression profiles in tumors, regardless of whether the gene expression profiles in normal samples available or not. Furthermore, by analyzing transcriptomic profiles in more than 30 cancer types, we demonstrated that DEPTH2 is competitive in characterizing the properties of ITH compared with most established algorithms, including DEPTH.

## Methods

### Datasets

We downloaded data of gene expression profiles (RSEM normalized), somatic mutation profiles (level 3), protein expression profiles (reverse phase protein array (RPPA) normalized), and clinical features for 33 TCGA cancer types from the genomic data commons data portal (https://portal.gdc.cancer.gov/). The 33 cancer types included adrenocortical carcinoma (ACC), bladder urothelial carcinoma (BLCA), breast invasive carcinoma (BRCA), cervical squamous cell carcinoma and endocervical adenocarcinoma (CESC), cholangiocarcinoma (CHOL), colon adenocarcinoma (COAD), lymphoid neoplasm diffuse large B-cell lymphoma (DLBC), esophageal carcinoma (ESCA), glioblastoma multiforme (GBM), head and neck squamous cell carcinoma (HNSC), kidney chromophobe (KICH), kidney renal clear cell carcinoma (KIRC), kidney renal papillary cell carcinoma (KIRP), acute myeloid leukemia (LAML), brain lower grade glioma (LGG), liver hepatocellular carcinoma (LIHC), lung adenocarcinoma (LUAD), lung squamous cell carcinoma (LUSC), mesothelioma (MESO), ovarian serous cystadenocarcinoma (OV), pancreatic adenocarcinoma (PAAD), pheochromocytoma and paraganglioma (PCPG), prostate adenocarcinoma (PRAD), rectum adenocarcinoma (READ), sarcoma (SARC), skin cutaneous melanoma (SKCM), stomach adenocarcinoma (STAD), testicular germ cell tumors (TGCT), thyroid carcinoma (THCA), thymoma (THYM), uterine corpus endometrial carcinoma (UCEC), uterine carcinosarcoma (UCS), and uveal melanoma (UVM). For each RSEM normalized gene expression value, we added it to 1 and then log2-transformed. We downloaded transcriptomic data (Affymetrix Human Genome U219 array) for cancer cell lines and drug sensitivities (IC50 values) of these cell lines to 265 compounds from the Genomics of Drug Sensitivity in Cancer (GDSC) project (https://www.cancerrxgene.org/downloads). In addition, we downloaded 34 validation datasets from three resources: GEO (https://www.ncbi.nlm.nih.gov/geo/), ArrayExpress (https://www.ebi.ac.uk/arrayexpress/), and UCSC (https://xenabrowser.net/datapages/). We also downloaded gene expression profiles and clinical data for four cancer cohorts receiving anti-PD-1/PD-L1/CTLA-4 immunotherapies, including the Allen (melanoma) [8], Snyder (melanoma) [9], Miao (clear cell renal cell carcinoma) [10], and Jung cohorts (non-small cell lung carcinoma) [11]. A summary of these datasets is presented in Additional file 1: Table S1.

### Algorithm for quantifying ITH without reference to normal controls

Given a normalized gene expression matrix containing *m* genes and *t* tumor samples, the DEPTH2 score of tumor sample *T* is defined as

$$\begin{aligned} & \sqrt {\frac{{ \sum \nolimits_{i = 1}^{m} \left( {z\left( {ex\left( {Gi,T} \right)} \right) - \frac{1}{m}\sum \nolimits_{i = 1}^{m} z\left( {ex\left( {Gi,T} \right)} \right)} \right)^{2} }}{m - 1}} \\ & = \sqrt {\frac{{ \sum \nolimits_{i = 1}^{m} \left( {\frac{{\left| {(ex\left( {Gi,T} \right) - \frac{1}{t} \sum \nolimits_{j = 1}^{t} ex\left( {Gi,TSj} \right)} \right|}}{{\sqrt {\frac{{\mathop \sum \nolimits_{j = 1}^{t} \left( {ex\left( {Gi,T} \right) - \frac{1}{t}\mathop \sum \nolimits_{j = 1}^{t} ex\left( {Gi,TSj} \right)} \right)^{2} }}{t - 1}}}} - \frac{1}{m}\mathop \sum \nolimits_{i = 1}^{m} \frac{{\left| {(ex\left( {Gi,T} \right) - \frac{1}{t}\mathop \sum \nolimits_{j = 1}^{t} ex\left( {Gi,TSj} \right)} \right|}}{{\sqrt {\frac{{ \sum \nolimits_{j = 1}^{t} \left( {ex\left( {Gi,T} \right) - \frac{1}{t} \sum \nolimits_{j = 1}^{t} ex\left( {Gi,TSj} \right)} \right)^{2} }}{t - 1}} }}} \right)^{2} }}{m - 1},} \\ z\left( {ex\left( {Gi,T} \right)} \right) & = \frac{{\left| {(ex\left( {Gi,T} \right) - \frac{1}{t}\sum \nolimits_{j = 1}^{t} ex\left( {Gi,TSj} \right)} \right|}}{SDi}, \\ SDi & = \sqrt {\frac{{\sum \nolimits_{j = 1}^{t} \left( {ex\left( {Gi,T} \right) - \frac{1}{t}\sum \nolimits_{j = 1}^{t} ex\left( {Gi,TSj} \right)} \right)^{2} }}{t - 1}} , \\ \end{aligned}$$

where *ex*(*x*, *y*) denotes gene *x* expression level in the tumor sample *y*.

**Fig. 1:**
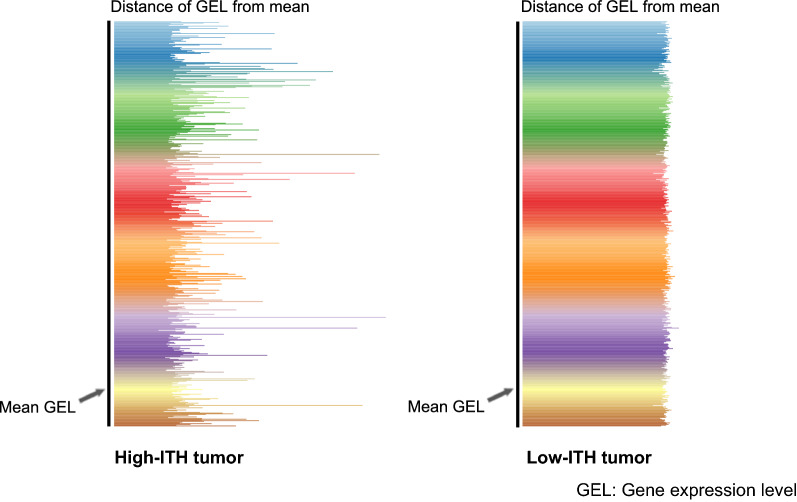
Illustration of two bulk tumors with high and low DEPTH2 scores, respectively

DEPTH2 calculated a tumor’s ITH level based on the standard deviations of absolute z-scored expression values of a set of genes in the tumor. In a tumor, if most genes show close absolute z-scored expression values, the tumor will likely have a low DEPTH2 score, namely low ITH level; otherwise, the tumor will have a relatively high DEPTH2 score. Therefore, the DEPTH2 score reflects the asynchronous level of gene expression alterations relative to the central tendency normalized by standard deviation in all tumors for all genes in the gene expression matrix (Fig. 1). We argued that the asynchronous level was determined by the heterogeneity level of gene expression profiles among different tumor cells within a tumor and thus reflects the ITH level at the mRNA level. The R package for the DEPTH2 algorithm is available at the website: https://github.com/XS-Wang-Lab/DEPTH2, under a GNU GPL open-source license.

### Evaluation of genomic instability and tumor purity

The tumor mutation burden (TMB) in a tumor was defined as the total number of somatic mutations in the tumor. We obtained copy number alteration (CNA) scores and homologous recombination deficiency (HRD) scores in TCGA cancers from the publication by Knijnenburg et al. [12]. We calculated tumor purity using the ESTIMATE [13] algorithm with the input of gene expression profiles.

### Survival analysis

We compared survival prognosis between higher-DEPTH2-score (> median) and lower-DEPTH2-score (< median) tumors in pan-cancer (overall survival (OS), disease-specific survival (DSS), disease-free interval (DFI), and progression-free interval (PFI)) and in individual cancer types (OS and disease-free survival (DFS)). We used Kaplan–Meier (KM) curves to display survival time differences and the log-rank test to assess the significance of survival time differences. The survival analyses were implemented using the function “survfit” in the R package “survival”.

### Gene-set enrichment analysis

We scored the enrichment levels of immune, stemness, and pathway signatures using the single-sample gene-set enrichment analysis (ssGSEA) [14]. The ssGSEA scored these features based on the expression levels of their marker or pathway genes. The ratio of two immune signatures in a tumor sample was the geometric mean expression level of all marker genes in an immune signature divided by that in another immune signature (log_2_-transformed). The marker or pathway gene sets are presented in Additional file 1: Table S2.

### ITH evaluated by other algorithms

We calculated MATH ITH scores using the function “math.score” [2] in the R package “maftools” with the input of “maf” files. We calculated ABSOLUTE ITH scores, namely ploidy scores, using the ABSOLUTE algorithm [15] with the input of “SNP6” files. Both “maf” and “SNP6” files were obtained from the genomic data commons data portal (https://portal.gdc.cancer.gov/). In addition, we obtained the ITH scores calculated by DEPTH [6], PhyloWGS [5] and tITH [7] from their associated publications.

### Statistical analysis

In evaluating correlations between ITH scores and other variables, we used the Spearman’s correlation and reported the correlation coefficient (*ρ*) and *P*-value. In comparisons of ITH scores among different classes, we used the one-sided Mann–Whitney *U* test for two classes and Kruskal–Wallis test for more than two classes. We used the Benjamini and Hochberg method [16] to calculate the false discovery rate (FDR) for adjusting for multiple tests. All statistical analyses were implemented in the R programming environment (version 4.0.2).

## Results

### DEPTH2 ITH correlates with unfavorable clinical outcomes in cancer

**Fig. 2:**
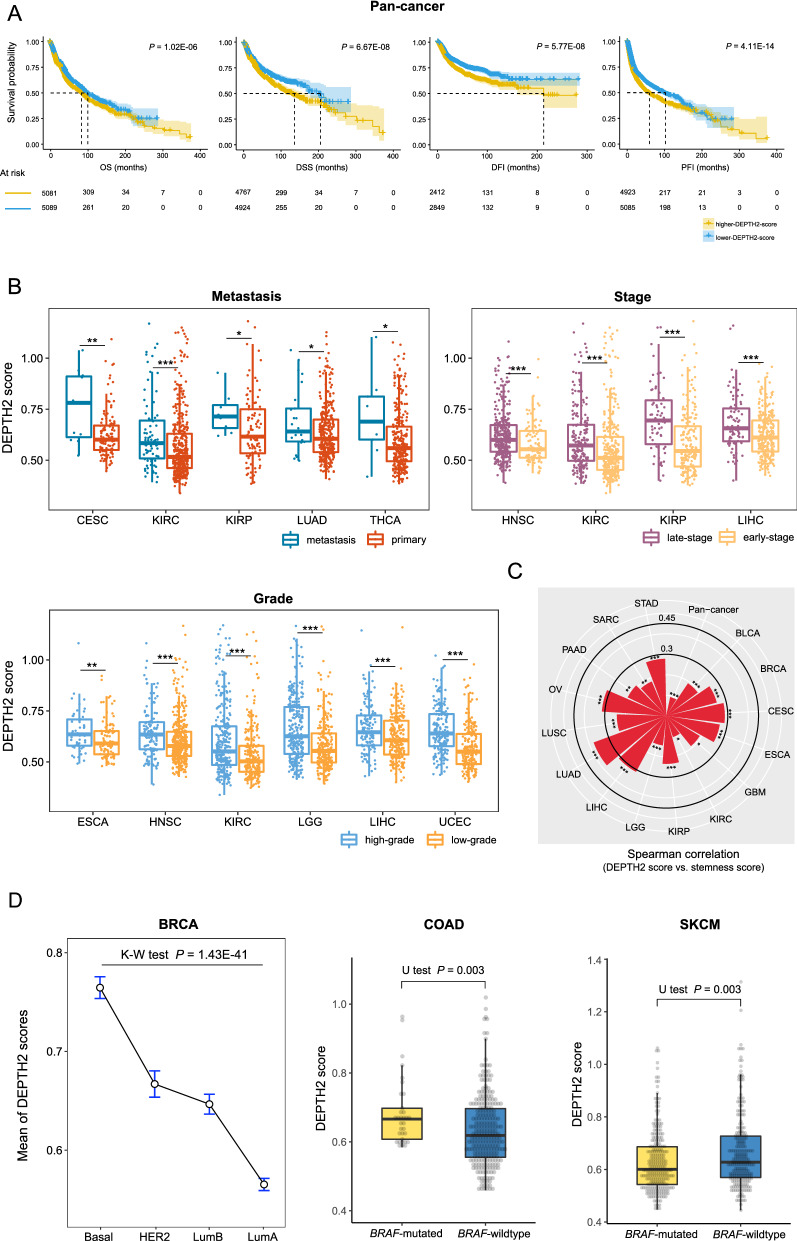
DEPTH2 ITH correlating with unfavorable clinical outcomes in cancer. **A** Kaplan–Meier curves showing that higher-DEPTH2-score (> median) tumors have more inferior survival than lower-DEPTH2-score (< median) tumors in pan-cancer. The log-rank test *P*-values are shown. OS: overall survival, DSS: disease-specific survival, PFI: progression-free interval, DFI: disease-free interval. **B** DEPTH2 scores are significantly higher in advanced versus non-advanced [metastatic versus primary, late-stage (stage III–IV) versus early-stage (stage I–II), and high-grade (G3–4) versus low-grade (G1–2)] tumors in diverse cancers (one-tailed Mann–Whitney *U* test, *P* < 0.05). **C** Significant positive correlations between DEPTH2 scores and tumor stemness scores in pan-cancer and in 15 individual cancer types (Spearman correlation, *P* < 0.05). **D** Comparisons of DEPTH2 scores among cancer subtypes. K–W test, Kruskal–Wallis test; *U* test, Mann–Whitney *U* test. *P < 0.05, **P < 0.01, ***P < 0.001

We found that higher DEPTH2 scores correlated with worse survival in pan-cancer (log-rank test, *P* = 1.02 × 10^–6^, 6.67 × 10^–8^, 5.77 × 10^–8^, and 4.11 × 10^–14^ for OS, DSS, DFI, and PFI, respectively) (Fig. 2A). In addition, higher DEPTH2 scores were associated with worse OS in 10 cancer types (ACC, BLCA, BRCA, COAD, KIRC, KIRP, LGG, SKCM, THCA, and UCEC), as well as worse DFS in 10 cancer types (ACC, COAD, KIRC, KIRP, LGG, LIHC, PCPG, SKCM, STAD, and UCEC) (log-rank test, *P* < 0.05) (Additional file 2: Fig. S1). It is consistent with that ITH has a negative association with prognosis in cancer patients [17]. Furthermore, we found that DEPTH2 scores were significantly higher in metastatic than in primary tumors in 5 cancer types (CESC, KIRC, KIRP, LUAD, and THCA) (one-tailed Mann–Whitney *U* test, *P* < 0.05) (Fig. 2B). Moreover, DEPTH2 scores were significantly higher in late-stage (stage III–IV) versus early-stage (stage I–II) tumors in 4 cancer types (HNSC, KIRC, KIRP, and LIHC) and significantly higher in high-grade (G3–4) versus low-grade (G1–2) tumors in 6 cancer types (ESCA, HNSC, KIRC, LGG, LIHC, and UCEC) (*P* < 0.05) (Fig. 2B). Tumor stemness indicates the stem cell-like properties displayed in a subpopulation of cancer cells, namely continuous self-renewal and extensive proliferation [18]. We found that DEPTH2 scores correlated positively with tumor stemness scores in 15 cancer types and correlated negatively with tumor stemness scores in 3 cancer types (*P* < 0.05) (Fig. 2C).

We also found that DEPTH2 scores were likely higher in more aggressive versus less aggressive cancer subtypes. For example, in BRCA, DEPTH2 scores followed the pattern: basal-like > HER2-enriched > luminal B > luminal A (Kruskal–Wallis test, *P* < 0.001) (Fig. 2D). In COAD, the *BRAF*-mutated subtype displayed significantly higher DEPTH2 scores than the *BRAF*-wildtype subtype (*P* = 0.003) (Fig. 2D). In contrast, in SKCM, the *BRAF*-mutated subtype displayed significantly lower DEPTH2 scores than the *BRAF*-wildtype subtype (*P* = 0.003) (Fig. 2D). Previous studies have showed that *BRAF* mutations are associated with a worse prognosis in colorectal cancer [19] and a better prognosis in melanoma [20].

Collectively, these results indicate that the DEPTH2 ITH level is an adverse prognostic factor in diverse cancers.

### DEPTH2 scores correlate positively with genomic instability in cancer

**Fig. 3:**
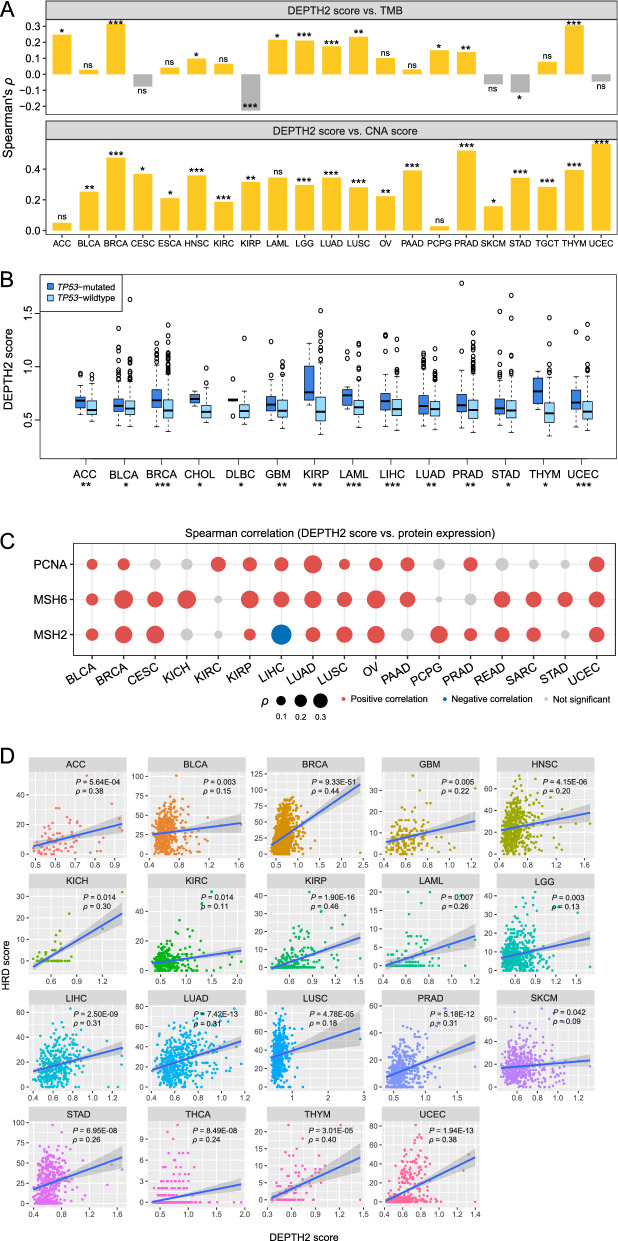
DEPTH2 ITH correlating positively with genomic instability in cancer. **A** DEPTH2 scores having significant positive correlations with tumor mutation burden (TMB) and copy number alteration (CNA) scores in multiple cancer types. The Spearman correlation coefficients (*ρ*) and *P* values are shown. TMB: the total somatic mutation count in the tumor. **B** DEPTH2 scores are significantly higher in *TP53*-mutated than in *TP53*-wildtype tumors in 14 cancer types (one-tailed Mann–Whitney *U* test, *P* < 0.05). **C** DEPTH2 scores having significant positive correlations with the expression of DNA mismatch repair proteins (MSH2, MSH6, and PCNA) in diverse cancers. **D** DEPTH2 scores having significant positive correlations with homologous recombination deficiency (HRD) scores in diverse cancers (Spearman correlation, *P* < 0.05). *P < 0.05, **P < 0.01, ***P < 0.001, *ns* not significant

Genomic instability may increase TMB and CNA [21]. We found that TMB had a positive correlation with DEPTH2 scores in 10 individual cancer types (*P* < 0.05) (Fig. 3A). In 18 cancer types, CNA scores correlated positively with DEPTH2 scores (*P* < 0.05) (Fig. 3A). Because p53 has a critical role in maintaining genomic stability, *TP53* mutations may promote genomic instability [22, 23]. We found that DEPTH2 scores were remarkably higher in *TP53*-mutated than in *TP53*-wildtype tumors in 14 cancer types (*P* < 0.05) (Fig. 3B). Further, DNA mismatch repair deficiency and HRD are important factors responsible for genomic instability in cancer [21]. We found that the expression levels of three DNA mismatch repair proteins MSH2, MSH6, and PCNA correlated positively with DEPTH2 scores in 12, 14, and 11 cancer types, respectively (*P* < 0.05) (Fig. 3C). Furthermore, DEPTH2 scores correlated positively with HRD scores [12] in pan-cancer (*ρ* = 0.05, *P* = 1.27 × 10^–6^) and in 19 individual cancer types (*P* < 0.05) (Fig. 3D). Taken together, these results suggest a strong positive association between the DEPTH2 ITH level and genomic instability in cancer.

### DEPTH2 scores correlate negatively with antitumor immune response

**Fig. 4:**
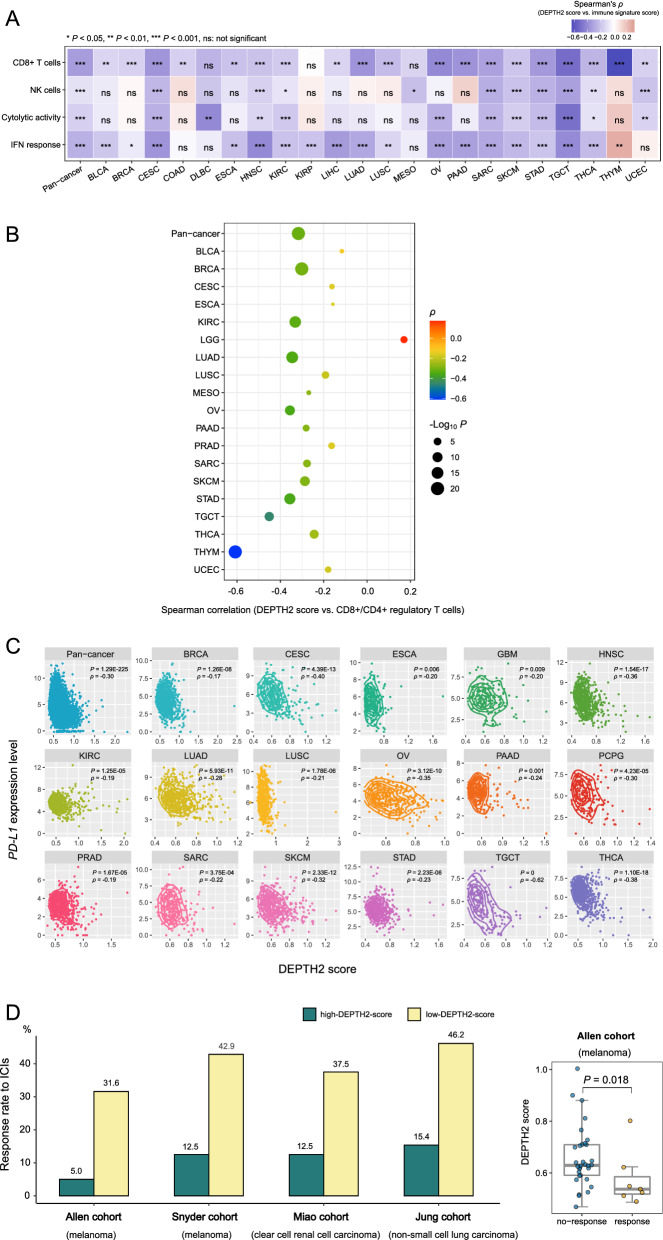
DEPTH2 ITH correlating negatively with antitumor immune response and immunotherapy response. Spearman correlations between DEPTH2 scores and the enrichment levels of antitumor immune signatures (CD8+ T cells, NK cells, immune cytolytic activity, and interferon (IFN) response) (**A**), ratios of immune-stimulatory/immune-inhibitory signatures (CD8+/CD4+ regulatory T cells) (**B**), and *PD-L1* expression levels (**C**) in pan-cancer and individual cancer types. **D** High-DEPTH2-score (> median) tumors show a lower response rate to immune checkpoint inhibitors (ICIs) than the low-DEPTH2-score (< median) tumors in four cancer cohorts receiving anti-PD-1/PD-L1/CTLA-4 immunotherapies, and DEPTH2 scores are significantly lower in the tumors responsive to ICIs than in the tumors not responsive to ICIs in the Allen cohort

ITH may inhibit antitumor immune response [1]. We analyzed the correlations between the enrichment levels of four immune signatures (CD8+ T cells, NK cells, immune cytolytic activity, and IFN response) and DEPTH2 scores in cancer. We found that the enrichment levels of these immune signatures had significant inverse correlations with DEPTH2 scores in pan-cancer (CD8+ T cells: *ρ* = -0.34, *P* = 1.36 × 10^–293^; NK cells: *ρ* = − 0.10, *P* = 3.28 × 10^–26^; immune cytolytic activity: *ρ* = − 0.16, *P* = 4.11 × 10^–63^; IFN response: *ρ* = − 0.27, *P* = 2.86 × 10^–181^) and in 19, 10, 11, and 17 cancer types, respectively (*P* < 0.05) (Fig. 4A). Moreover, the ratios of immune-stimulatory/immune-inhibitory signatures (CD8+/CD4+ regulatory T cells) also showed significant inverse correlations with DEPTH2 scores in pan-cancer (*ρ* = − 0.32, *P* = 2.05 × 10^–256^) and in 18 cancer types (*P* < 0.05) (Fig. 4B). These results suggest a negative association between the DEPTH2 ITH level and antitumor immunity, supporting previous findings that ITH inhibits antitumor immune response [1].

Interestingly, DEPTH2 scores were inversely associated with *PD-L1* expression levels in pan-cancer (*P* = 1.29 × 10^–225^, *ρ* = − 0.30) and in 17 individual cancer types (*P* < 0.05) (Fig. 4C). Because both tumor-infiltrating lymphocyte enrichment [24] and PD-L1 expression [25] are positive predictors for anti-PD-1/PD-L1/CTLA-4 immunotherapy response and DEPTH2 scores likely correlated negatively with both of them, we anticipated that the tumors with high DEPTH2 ITH would respond worse to immune checkpoint inhibitors (ICIs) versus the tumors with low DEPTH2 ITH. As expected, we found that the high-DEPTH2-score (> median) tumors showed a lower response rate to ICIs than the low-DEPTH2-score (< median) tumors in four cancer cohorts receiving anti-PD-1/PD-L1/CTLA-4 immunotherapies (Allen cohort: 5% versus 31.6%; Snyder cohort: 12.5% versus 42.9%; Miao cohort: 12.5% versus 37.5%; Jung cohort: 15.4% versus 46.2%) (Fig. 4D). In addition, the tumors responsive to ICIs had significantly lower DEPTH2 scores than the tumors not responsive to ICIs in the Allen cohort (*P* = 0.018) (Fig. 4D). These results support that increased DEPTH2 ITH may inhibit immunotherapy response in cancer.

### DEPTH2 scores correlate positively with tumor purity

**Fig. 5:**
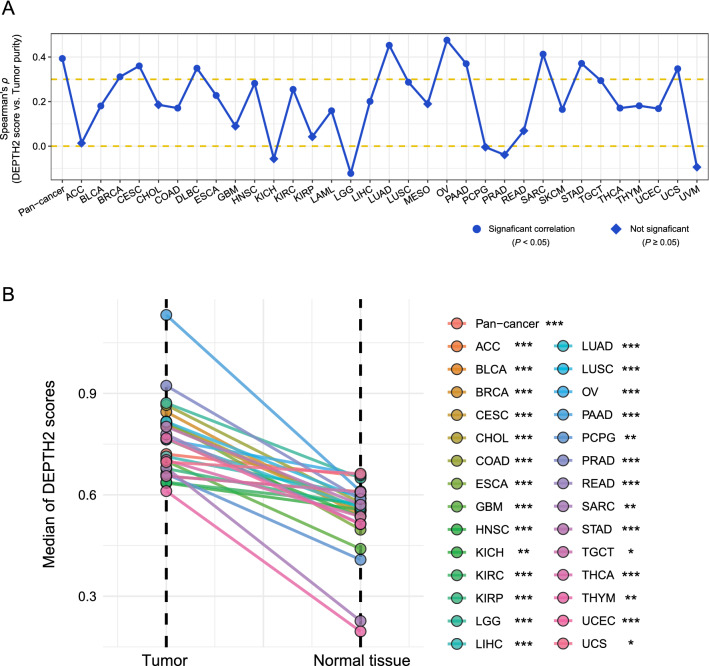
DEPTH2 ITH correlating positively with tumor purity. **A** Significant positive correlations between DEPTH2 scores and tumor purity in pan-cancer and in 22 individual cancer types. **B** Significantly higher DEPTH2 scores versus normal controls in pan-cancer and in all individual cancer types with normal controls available. The one-tailed Mann–Whitney *U* test *P* values are indicated. *P < 0.05, **P < 0.01, ***P < 0.001

Notably, DEPTH2 scores had significant positive correlations with tumor purity in pan-cancer (*ρ* = 0.39, *P* < 0.001) and in 22 cancer types (*P* < 0.05) (Fig. 5A). It implies that the DEPTH2 ITH level increases with the increase of tumor purity. To further show that the DEPTH2 measure does indeed represent ITH between tumor cells within a bulk tumor, we calculated the DEPTH2 scores in normal controls for 30 cancer types with related data available. As expected, DEPTH2 scores were significantly lower in normal controls than in tumor samples in pan-cancer (*P* = 1.63 × 10^–142^) and in all the 28 cancer types (*P* < 0.05) (Fig. 5B).

To correct for the impact of tumor purity on the relationship between DEPTH2 scores and genomic instability, we divided DEPTH2 scores by proportions of tumor cells in bulk tumors, which were obtained from the TCGA pathological slides data, termed tumor purity-adjusted DEPTH2 scores. Notably, the tumor purity-adjusted DEPTH2 scores showed significant positive correlations with TMB, CNA, and HRD in 5, 11, and 12 cancer types, respectively (*P* < 0.05) (Additional file 3: Fig. S2A); they correlated inversely with the enrichment scores of CD8+ T cells, NK cells, immune cytolytic activity, and IFN response in 14, 9, 10, and 13 cancer types, respectively (*P* < 0.05) (Additional file 3: Fig. S2B); they correlated positively with stemness scores in 11 cancer types (Additional file 3: Fig. S2C). Moreover, the tumor purity-adjusted DEPTH2 scores were significantly higher in late-stage versus early-stage tumors, in high-grade versus low-grade tumors, and in metastatic versus primary tumors in 8, 6, and 4 cancer types, respectively (Additional file 3: Fig. S2D). Meanwhile, the tumor purity-adjusted DEPTH2 scores still had significant negative correlations with survival prognosis in diverse cancer types (Additional file 3: Fig. S2E). These results suggest that the significant associations of DEPTH2 scores with antitumor immune signatures, genomic instability, and clinical outcomes in cancer are independent of tumor purity.

### Proteins with significant expression correlations with DEPTH2 ITH

**Fig. 6:**
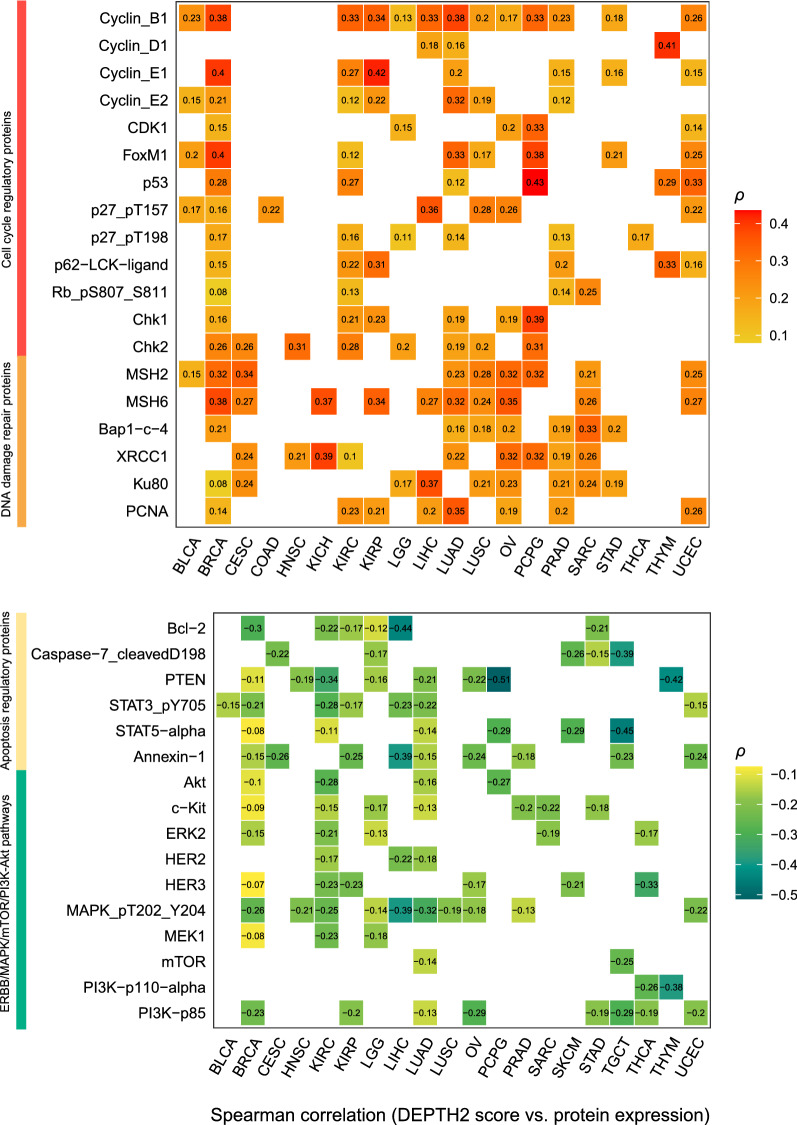
Proteins having significant expression correlations with DEPTH2 ITH in diverse cancers

We found 50 proteins having significantly positive expression correlations with DEPTH2 scores in at least 5 cancer types (FDR < 0.05) (Additional file 1: Table S3). Notably, many of these proteins were involved in the regulation of cell cycle, including Cyclin_B1, Cyclin_D1, Cyclin_E1, Cyclin_E2, CDK1, FoxM1, p53, p27, p62, Rb, Chk1, and Chk2 (Fig. 6). It suggests a positive association between cell cycle activity and DEPTH2 ITH. Several DNA damage repair proteins showed significantly positive expression correlations with DEPTH2 scores, including MSH2, MSH6, BAP1, XRCC1, Ku80, and PCNA (Fig. 6). In contrast, 48 proteins showed negative expression correlations with DEPTH2 scores in at least 5 cancer types (FDR < 0.05) (Additional file 1: Table S3). Notably, the 48 proteins included many apoptosis regulatory proteins, such as Bcl-2, Caspase-7, PTEN, STAT3, STAT5, and Annexin-1 (Fig. 6). It suggests a negative association between apoptotic activity and DEPTH2 ITH. In addition, many of the 48 proteins are involved in the ERBB/MAPK/mTOR/PI3K-Akt pathways, such as Akt, c-Kit, ERK2, HER2, HER3, MAPK, MEK1, mTOR, and PI3K (Fig. 6). Overall, these results are justified since cancer cell proliferation can promote the diversity of cancer cell phenotypes and thus the DEPTH2 ITH.

### DEPTH2 scores are associated with drug response in cancer

**Fig. 7:**
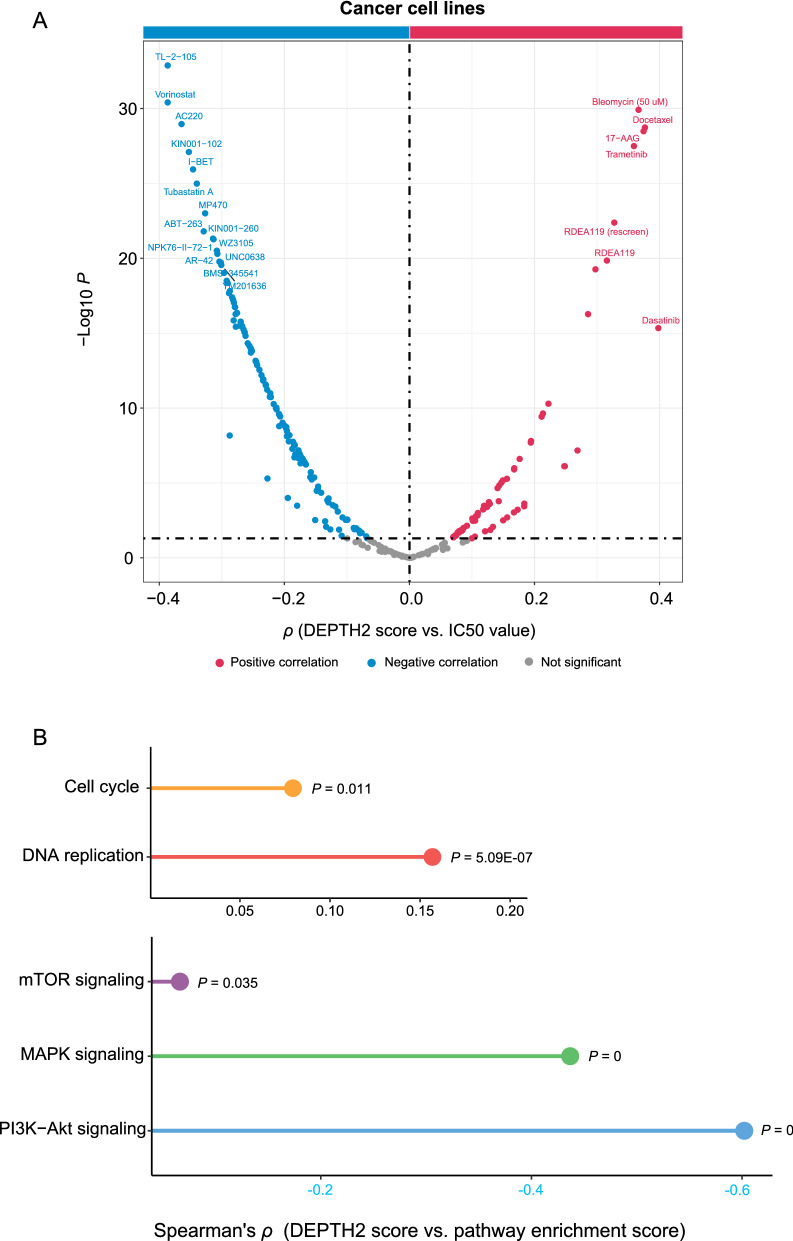
Compounds with significant correlations of drug sensitivity with DEPTH2 scores in cancer cell lines and their target pathways. **A** Of 265 antitumor compounds from the Genomics of Drug Sensitivity in Cancer (GDSC) project (https://www.cancerrxgene.org), 131 show a significant inverse correlation of IC50 values with DEPTH2 scores versus 68 showing a significant positive correlation (Spearman correlation, P < 0.05). The compounds whose IC50 values have a correlation of |*ρ*| > 0.3 with DEPTH2 scores are given. **B** The pathways having significant correlations of enrichment scores with DEPTH2 scores and being targeted by the compounds with significant correlations of drug sensitivity (IC50 values) with DEPTH2 scores in cancer cell lines. The Spearman correlation coefficients (*ρ*) and *P*-values are shown

In 265 antitumor compounds from the Genomics of Drug Sensitivity in Cancer (GDSC) project (https://www.cancerrxgene.org), 199 (75%) displayed significant correlations of drug sensitivity (IC50 values) with DEPTH2 scores in cancer cell lines (*P* < 0.05) (Additional file 1: Table S4). It suggests that the DEPTH2 ITH correlates with drug response for a wide range of anticancer drugs. Interestingly, among the 199 compounds, 131 had a significant inverse correlation of IC50 values with DEPTH2 scores versus 68 showing a significant positive correlation (Fig. 7A and Additional file 1: Table S4). It indicated that with the increase of the DEPTH2 ITH level, to many compounds, the sensitivity of cancer cells could increase or decrease. Interestingly, among the 131 compounds whose IC50 values correlated inversely with DEPTH2 scores, many targeted the cell cycle pathways, including NPK76-II-72-1, KIN001-270, THZ-2-102-1, PHA-793887, AT-7519, THZ-2-49 and CCT007093. In addition, two compounds (Methotrexate and Temozolomide) targeted the DNA replication pathway. These results are justified since DEPTH2 scores showed significant positive correlations with the enrichment scores of the cell cycle and DNA replication pathways in cancer cell lines (*P* < 0.05) (Fig. 7B). In contrast, among the 68 compounds whose IC50 values correlated positively with DEPTH2 scores, many targeted the pathways upregulated in low-DEPTH2-score tumors, such as AZ628, CI-1040, RDEA119, PD-0325901, AZD6244 and Trametinib targeted the MAPK signaling pathway, and KIN001-102, CAL-101, GSK2126458, OSI-027, PIK-93, YM201636, GSK690693, MK-2206, AZD8055 and PF-4708671 targeted the PI3K/mTOR signaling pathway. Again, these results are reasonable in terms of the significant negative correlations between DEPTH2 scores and the enrichment scores of the MAPK, PI3K-Akt, and mTOR signaling pathways in cancer cell lines (*P* < 0.05) (Fig. 7B).

### Associations between different ITH evaluation algorithms

**Fig. 8:**
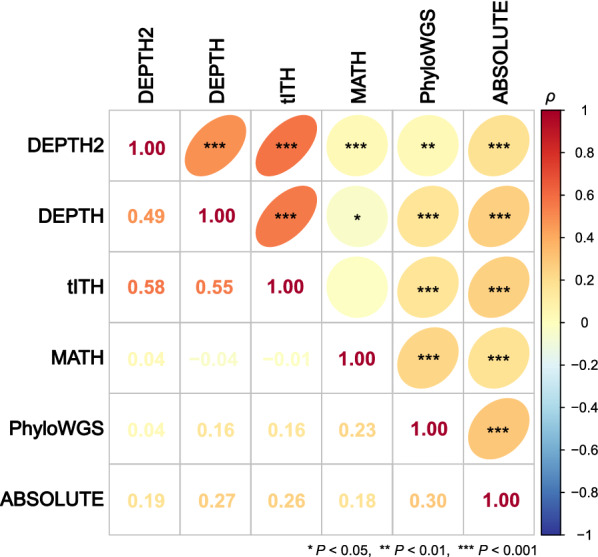
Pairwise correlations between ITH scores inferred by six different algorithms in pan-cancer. The Spearman correlation coefficients (*ρ*) and *P*-values are shown

We explored associations between DEPTH2 ITH scores and ITH scores by other five algorithms, including MATH [2], PhyloWGS [5], ABSOLUTE [15], DEPTH [6], and tITH [7]. The five algorithms evaluate ITH at different molecular levels, with MATH, PhyloWGS, and ABSOLUTE at the DNA level, and DEPTH2 and tITH at the mRNA level. In pan-cancer, DEPTH2 scores had significant positive correlations with ITH scores by all the other algorithms (*P* < 0.01) and had the strongest correlations with tITH (*ρ* = 0.58) and DEPTH2 scores (*ρ* = 0.49) (Fig. 8). DEPTH2 scores had the weakest correlations with MATH and PhyloWGS scores in pan-cancer (*ρ* = 0.04). In most individual cancer types, DEPTH2 scores showed significant positive correlations with DEPTH and tITH scores (*P* < 0.05) (Additional file 1: Table S5). Particularly, DEPTH2 scores had strong positive correlations with DEPTH scores in 20 cancer types and with tITH scores in 5 cancer types (*ρ* > 0.5). However, there was no any individual cancer type in which DEPTH2 scores showed a strong positive correlation with ITH scores by MATH, PhyloWGS, or ABSOLUTE. These results indicate that DEPTH2 scores have stronger correlations with ITH scores by mRNA-based algorithms than by DNA-based algorithms.

### Comparisons of DEPTH2 with other ITH evaluation algorithms

We further compared DEPTH2 with the five ITH evaluation algorithms in their correlations with clinical features, genomic instability, antitumor immune response, and tumor purity in the 33 TCGA cancer types.

**Fig. 9:**
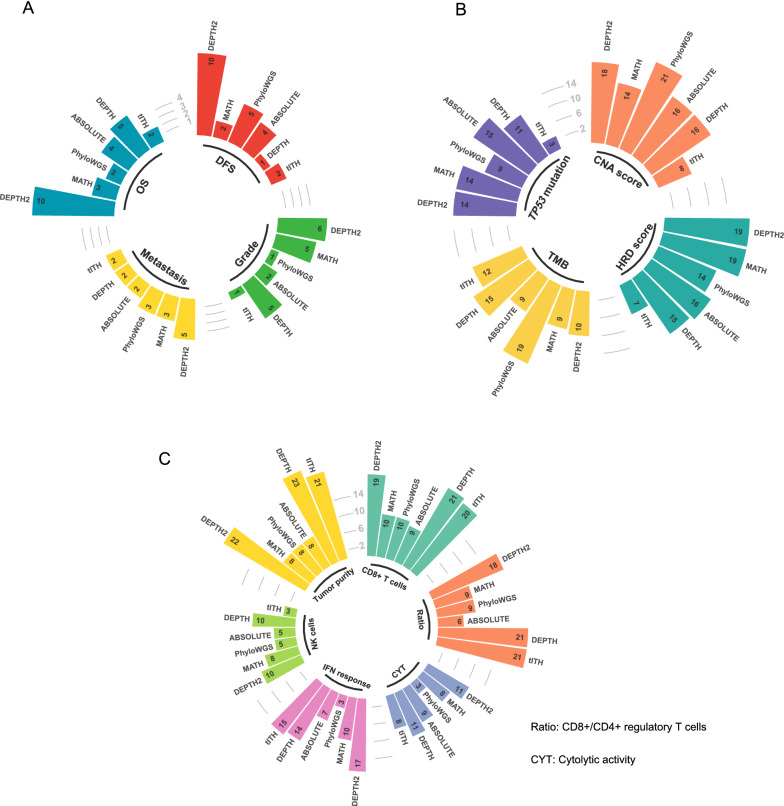
Comparisons of DEPTH2 with other five ITH evaluation algorithms. The numbers of cancer types in which ITH scores by each algorithm display significant correlations with clinical or pathological features (**A**), genomic instability (**B**), as well as antitumor immune response and tumor purity (**C**) among the 33 TCGA cancer types are shown

ITH scores by MATH, PhyloWGS, ABSOLUTE, DEPTH, and tITH were negatively correlated with OS time in 3, 2, 4, 5, and 2 cancer types, respectively, compared to DEPTH2 in 10 cancer types (log-rank test, *P* < 0.05) (Fig. 9A). Additionally, ITH scores by MATH, PhyloWGS, ABSOLUTE, DEPTH, and tITH were negatively correlated with DFS time in 2, 5, 4, 1, and 2 cancer types, respectively, compared to DEPTH2 in 10 cancer types (*P* < 0.05) (Fig. 9A). ITH scores by MATH, PhyloWGS, ABSOLUTE, DEPTH, and tITH were significantly higher in metastatic than in primary tumors in 3, 3, 2, 2, and 2 cancer types, respectively, compared to DEPTH2 in 5 cancer types (*P* < 0.05) (Fig. 9A). Furthermore, ITH scores by MATH, PhyloWGS, ABSOLUTE, DEPTH, and tITH were significantly higher in high-grade than in low-grade tumors in 5, 1, 2, 5, and 1 cancer types, respectively, compared to DEPTH2 in 6 cancer types (*P* < 0.05) (Fig. 9A). Overall, these results suggest that the DEPTH2 ITH has a stronger association with unfavorable clinical outcomes in cancer than the other algorithms’ ITH.

We found that TMB had a positive correlation with ITH scores by MATH, PhyloWGS, ABSOLUTE, DEPTH, and tITH in 9, 19, 9, 15, and 12 cancer types, respectively, compared to DEPTH2 in 10 cancer types (*P* < 0.05) (Fig. 9B). CNA scores correlated positively with ITH scores by MATH, PhyloWGS, ABSOLUTE, DEPTH, and tITH in 14, 21, 16, 16, and 8 cancer types, respectively, compared to DEPTH2 in 18 cancer types (*P* < 0.05) (Fig. 9B). Furthermore, HRD scores had a positive correlation with ITH scores by MATH, PhyloWGS, ABSOLUTE, DEPTH, and tITH in 19, 14, 16, 15, and 7 cancer types, respectively, compared to DEPTH2 in 19 cancer types (*P* < 0.05) (Fig. 9B). In addition, *TP53* mutations were associated with higher ITH scores by MATH, PhyloWGS, ABSOLUTE, DEPTH, and tITH in 14, 9, 15, 11, and 3 cancer types, respectively, compared to DEPTH2 in 14 cancer types (*P* < 0.05) (Fig. 9B). Collectively, these results indicate that the association between the DEPTH2 ITH and genomic instability is stronger than or comparable with that between the other algorithms’ ITH and genomic instability.

The immune cytolytic activity scores showed a significant negative correlation with ITH scores by MATH, PhyloWGS, ABSOLUTE, DEPTH, and tITH in 8, 3, 9, 11, and 8 cancer types, respectively, compared to DEPTH2 in 11 cancer types (*P* < 0.05) (Fig. 9C). The IFN response scores were significantly and negatively correlated with ITH scores by MATH, PhyloWGS, ABSOLUTE, DEPTH, and tITH in 10, 3, 7, 14, and 15 cancer types, respectively, compared to DEPTH2 in 17 cancer types (*P* < 0.05) (Fig. 9C). The enrichment scores of NK cells were significantly and negatively correlated with ITH scores by MATH, PhyloWGS, ABSOLUTE, DEPTH, and tITH in 8, 5, 5, 10, and 3 cancer types, respectively, compared to DEPTH2 in 10 cancer types (*P* < 0.05) (Fig. 9C). The enrichment scores of CD8+ T cells had a significant negative correlation with ITH scores by MATH, PhyloWGS, ABSOLUTE, DEPTH, and tITH in 10, 10, 9, 21, and 20 cancer types, respectively, compared to DEPTH2 in 19 cancer types (*P* < 0.05) (Fig. 9C). Additionally, the ratios of CD8+/CD4+ regulatory T cells displayed a significant negative correlation with ITH scores by MATH, PhyloWGS, ABSOLUTE, DEPTH, and tITH in 9, 9, 6, 21, and 21 cancer types, respectively, compared to DEPTH2 in 18 cancer types (*P* < 0.05) (Fig. 9C). These results indicate that compared to the DNA-based ITH evaluation algorithms (MATH, PhyloWGS, and ABSOLUTE), DEPTH2 ITH is significantly and negatively correlated with the enrichment of antitumor immunes in many more cancer types. Tumor purity had a significant positive correlation with ITH scores by MATH, PhyloWGS, ABSOLUTE, DEPTH, and tITH in 8, 8, 8, 23, and 21 cancer types, respectively, compared to DEPTH2 in 22 cancer types (*P* < 0.05) (Fig. 9C). Again, it indicates that DEPTH2 is more likely to capture the ITH among tumor cells than the DNA-based algorithms.

Altogether, these results indicate that DEPTH2 is superior to or comparable with the other algorithms in characterizing ITH.

### Evaluation of DEPTH2 ITH in other datasets

**Fig. 10:**
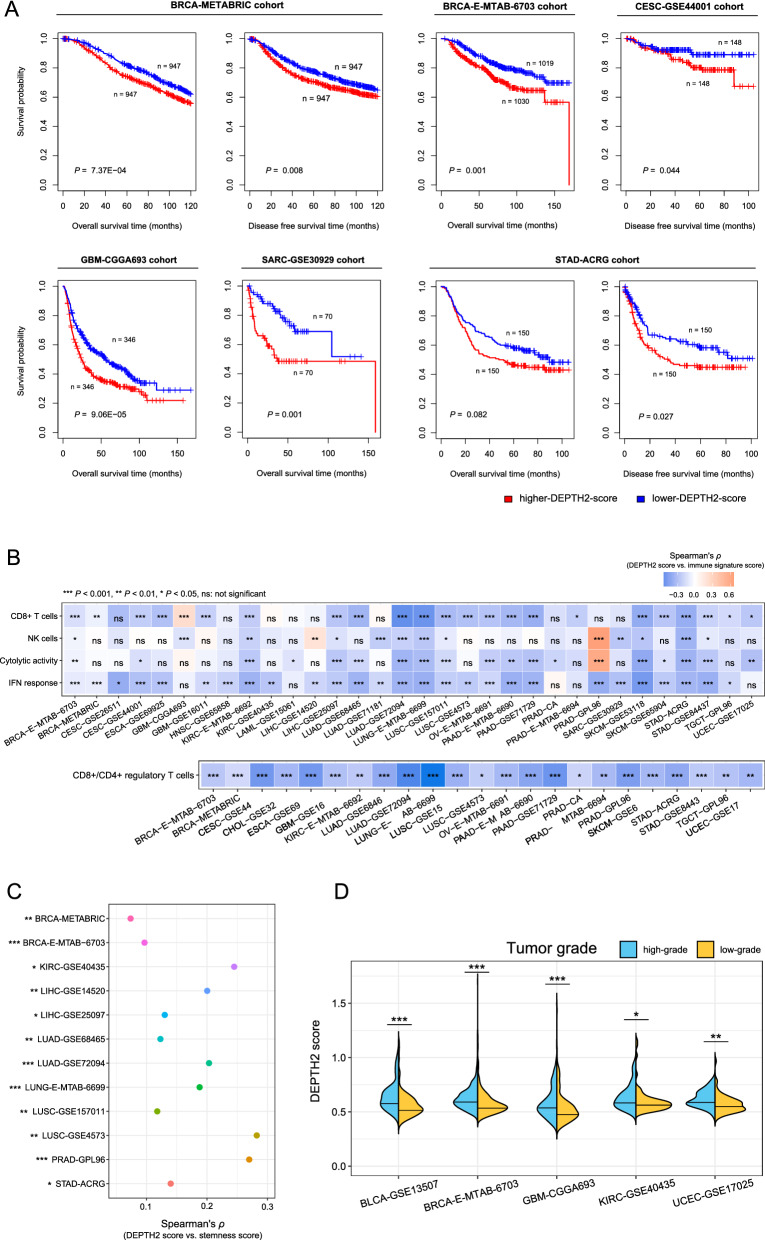
Validation of DEPTH2 ITH in other datasets. **A** Kaplan–Meier curves showing that higher-DEPTH2-score (> median) tumors have worse survival than lower-DEPTH2-score (< median) tumors in multiple validation datasets. The log-rank test *P*-values are shown. OS: overall survival, DFS: disease-free survival. DEPTH2 scores having significant positive correlations with antitumor immune signatures (**B**) and tumor stemness scores (**C**) in multiple validation datasets. **D** DEPTH2 scores are significantly higher in high-grade than in low-grade tumors in 5 validation datasets

Besides the TCGA cancers, we applied DEPTH2 to evaluate ITH levels in other 34 cancer transcriptomic datasets. These datasets were produced by different platforms and involved varied numbers of genes. In two breast cancer datasets E-MTAB-6703 (sample size n = 2302) and METABRIC (n = 1980), higher DEPTH2 scores (> median) were associated with worse OS and DFS (log-rank test, *P* < 0.01). In a gastric cancer dataset ACRG (n = 300), higher DEPTH2 scores were associated with worse DFS (log-rank test, *P* = 0.03). In some other datasets, such as GSE30929 (SARC, n = 140), CGGA693 (GBM, n = 693), and GSE44001 (CESC, n = 300), higher DEPTH2 scores were associated with worse OS or DFS (log-rank test, *P* < 0.05) (Fig. 10A). Moreover, DEPTH2 scores correlated negatively with the enrichment scores of CD8+ T cells, NK cells, immune cytolytic activity, and IFN response in 23, 12, 18, and 27 cancer types, respectively (*P* < 0.05) (Fig. 10B). In 23 datasets, DEPTH2 scores correlated negatively with the ratios of CD8+/CD4+ regulatory T cells (*P* < 0.05) (Fig. 10B). In 12 datasets, DEPTH2 scores correlated positively with tumor stemness scores (*P* < 0.05) (Fig. 10C). In addition, in 9 datasets with the tumor grade data available, DEPTH2 scores were significantly higher in high-grade than in low-grade tumors in 5 datasets (*P* < 0.02) (Fig. 10D). Altogether, these results confirmed the findings in TCGA datasets, supporting the reliability and robustness of DEPTH2 in measuring ITH.

## Discussion

In this study, we proposed a new mRNA-based ITH evaluation algorithm (DEPTH2), which is an improved version of DEPTH we developed previously [6]. One major advantage of DEPTH2 over DEPTH and other ITH evaluation algorithms is that DEPTH2 evaluates ITH without reference to normal controls. Hence, DEPTH2 should have a wider spectrum of applications in measuring ITH in comparison to DEPTH and most other algorithms. Furthermore, our data showed that DEPTH2 ITH was associated with tumor progression, unfavorable prognosis, genomic instability, reduced antitumor immunity and immunotherapy response, and altered drug response in diverse cancers. It suggests that DEPTH2 is effective in measuring ITH because the DEPTH2 metric reflects the common properties of ITH [1]. Moreover, our data suggest that DEPTH2 ITH likely has a stronger association with unfavorable clinical outcomes (e.g., survival prognosis) in cancer than the ITH evaluated by other algorithms. In characterizing other properties of ITH, such as its associations with genomic instability and antitumor immune evasion, DEPTH2 also displays competitive performance versus other algorithms.

DEPTH2 evaluates ITH levels based on the standard deviations of absolute z-scored transcriptome levels in tumors. The closer the absolute z-scored expression values, the lower the DEPTH2 score likely in the tumor. Thus, to a great degree, the DEPTH2 score indicates the asynchronous level of transcriptome alterations relative to the central tendency in a tumor. The asynchrony of transcriptome alterations in a tumor is associated with the heterogeneity of gene expression profiles among different tumor cells constituting the tumor. This is the rationale for DEPTH2 to evaluate the ITH level at the mRNA level.

Nevertheless, DEPTH2 has several limitations. First, because bulk tumors often involve non-tumor components, the ITH evaluated by DEPTH2 is likely confounded by non-tumor cells. To overcome this limitation, introducing the variable of tumor purity in the algorithm could be a solution. In addition, an investigation of the DEPTH2 algorithm in single-cell transcriptomes could eliminate the confounding effect of tumor purity. Second, due to differences in RNA-Seq or DNA microarray technology, read mapping, gene expression quantification methods, and the expression values of different genes, there could be large scale deviations between gene expression values that could have an impact on the generalization of DEPTH2 to a wide variety of data resources. To overcome this limitation, additional normalization strategies for gene expression values are needed, such as min–max scaling. In addition, nonparametric transformations of gene expression values could be a solution. We plan to further improve the DEPTH2 algorithm using these strategies in the future.

## Conclusions

DEPTH2 is a new algorithm evaluating ITH levels at the mRNA level. DEPTH2 is superior to or comparable with other methods in characterizing the properties of ITH and is expected to have a wider spectrum of applications in measuring ITH in comparison to most other algorithms. The DEPTH2 ITH may provide new insights into cancer biology, as well as potentially valuable markers for cancer diagnosis and treatment.

## Supplementary Information

**Additional file 1: Table S1.** A summary of the datasets used in this study. **Table S2.** The marker or pathway genes of immune signatures, stemness, and pathways. **Table S3.** Proteins with significant expression correlations with DEPTH2 scores in at least 5 cancer types. **Table S4.** Spearman correlations between DEPTH2 score and drug sensitivity (IC50 values) of cancer cell lines to 265 compounds. **Table S5.** Correlations between ITH scores inferred by six different algorithms within individual cancer types.

**Additional file 2: Figure S1.** Kaplan–Meier curves showing that higher-DEPTH2-score (> median) tumors have more inferior overall survival and disease-specific survival than lower-DEPTH2-score (< median) tumors in 10 and 10 individual cancer types, respectively. The log-rank test *P*-values are shown.

**Additional file 3: Figure S2.** Associations of tumor purity-adjusted DEPTH2 ITH with genomic instability, antitumor immune signatures, tumor progressive phenotypes, and survival prognosis in cancer. (A) Significant positive correlations between tumor purity-adjusted DEPTH2 scores and genomic instability features (TMB, CNA, and HRD) in diverse cancers. (B) Significant negative correlations between tumor purity-adjusted DEPTH2 scores and antitumor immune signatures (CD8+ T cells, NK cells, immune cytolytic activity, and IFN response) in diverse cancers. (C) Significant positive correlations between tumor purity-adjusted DEPTH2 scores and tumor stemness scores in 11 cancer types. (D) DEPTH2 scores are significantly higher in advanced versus non-advanced [late-stage (stage III–IV) versus early-stage (stage I–II), high-grade (G3–4) versus low-grade (G1–2), and metastatic versus primary] tumors in diverse cancers (one-tailed Mann–Whitney *U* test, *P* < 0.05). (E) Kaplan–Meier curves showing that the tumors with higher tumor purity-adjusted DEPTH2 scores (> median) have more inferior overall survival and disease-specific survival than the tumors with lower tumor purity-adjusted DEPTH2 scores (< median) in 5 and 6 individual cancer types, respectively. The log-rank test *P*-values are shown.

## Data Availability

All data supporting the findings of this study are available within the paper and its supplementary information. The R package for the DEPTH2 algorithm and the DEPTH2 scores of all cancer patients in 33 TCGA cancer types are available at: https://github.com/XS-Wang-Lab/DEPTH2.
