# Supplementary figures and images for: DEPTH2: an mRNA-based algorithm to evaluate intratumor heterogeneity without reference to normal controls
Source: J Transl Med. 2022 Apr 1;20:150. doi: 10.1186/s12967-022-03355-1 (PMC8974098; doi:10.1186/s12967-022-03355-1)

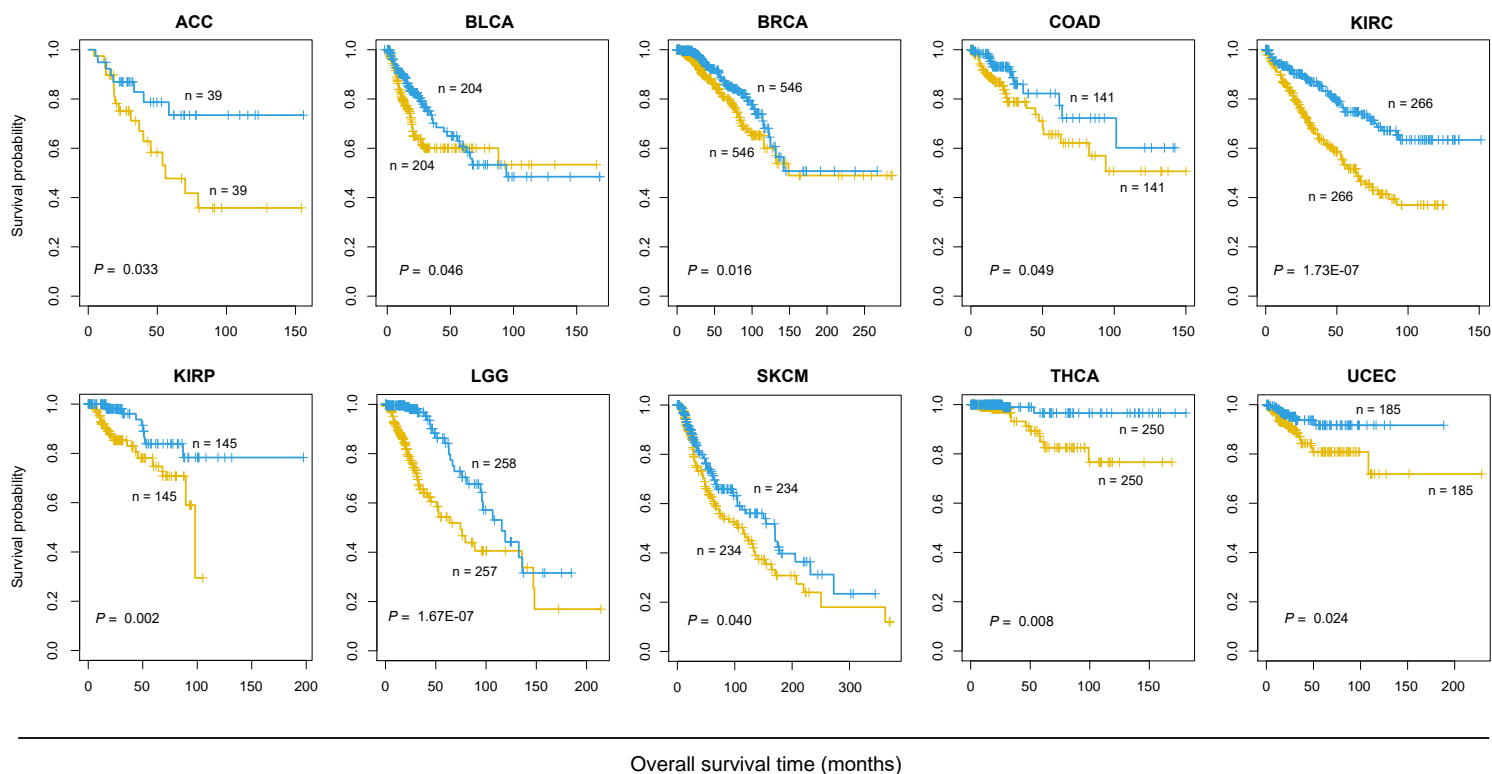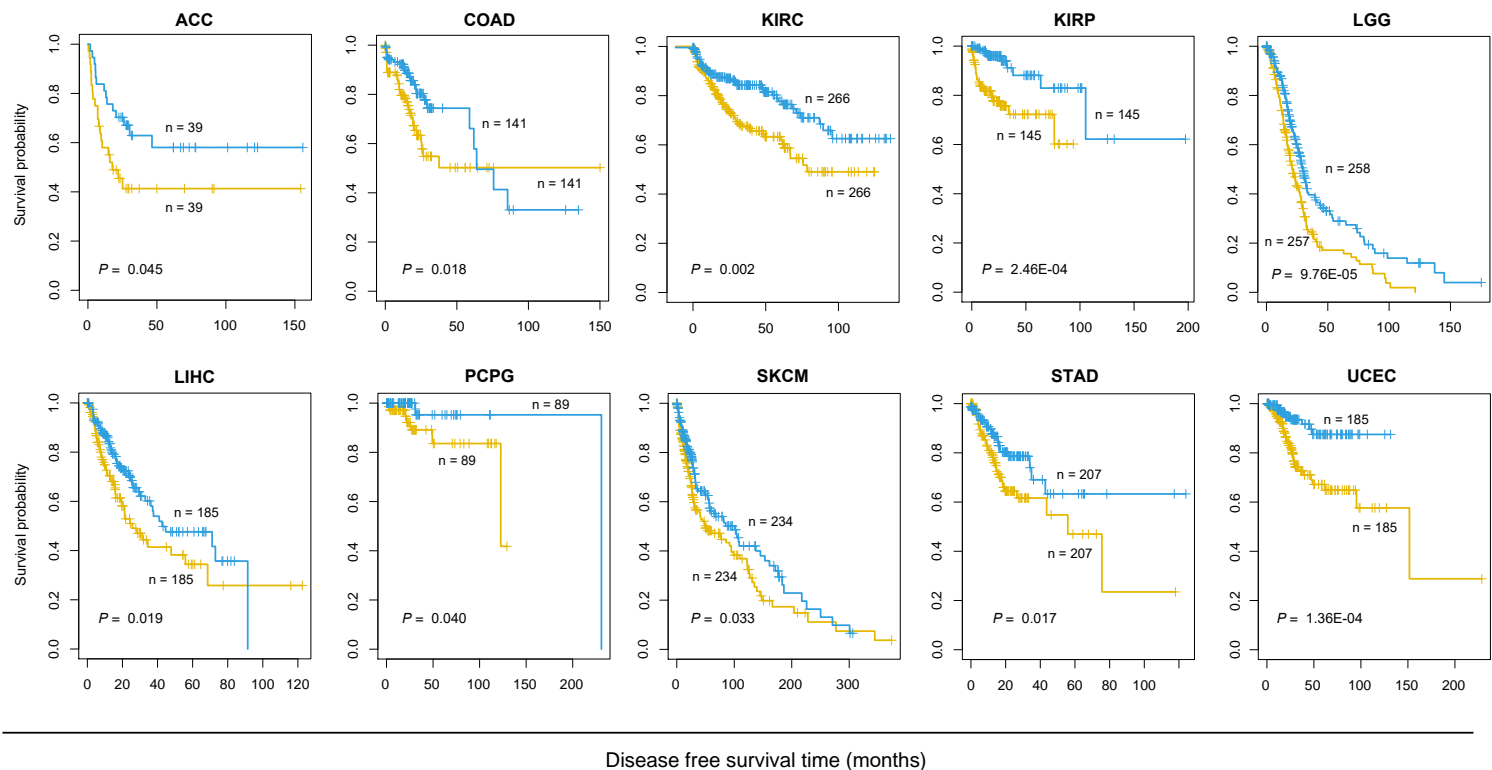

■ higher-DEPTH2-score  
■ lower-DEPTH2-score

Supplement: Supplementary file 2 — Additional file 2: Figure S1. Kaplan–Meier curves showing that higher-DEPTH2-score (> median) tumors have more inferior overall survival and disease-specific survival than lower-DEPTH2-score (< median) tumors in 10 and 10 individual cancer types, respectively. The log-rank test P-values are shown. [file 12967_2022_3355_MOESM2_ESM.pdf]

A

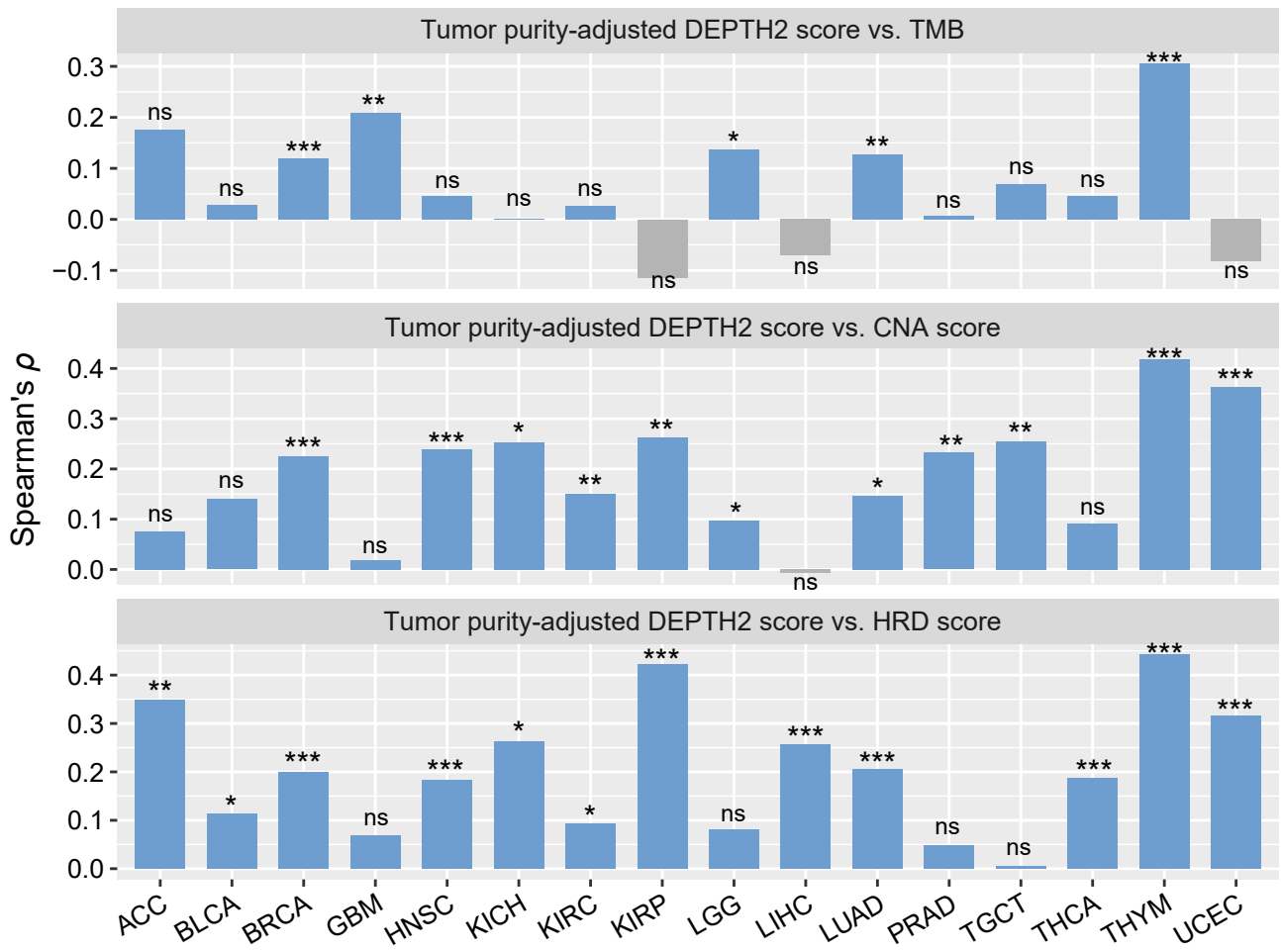

B

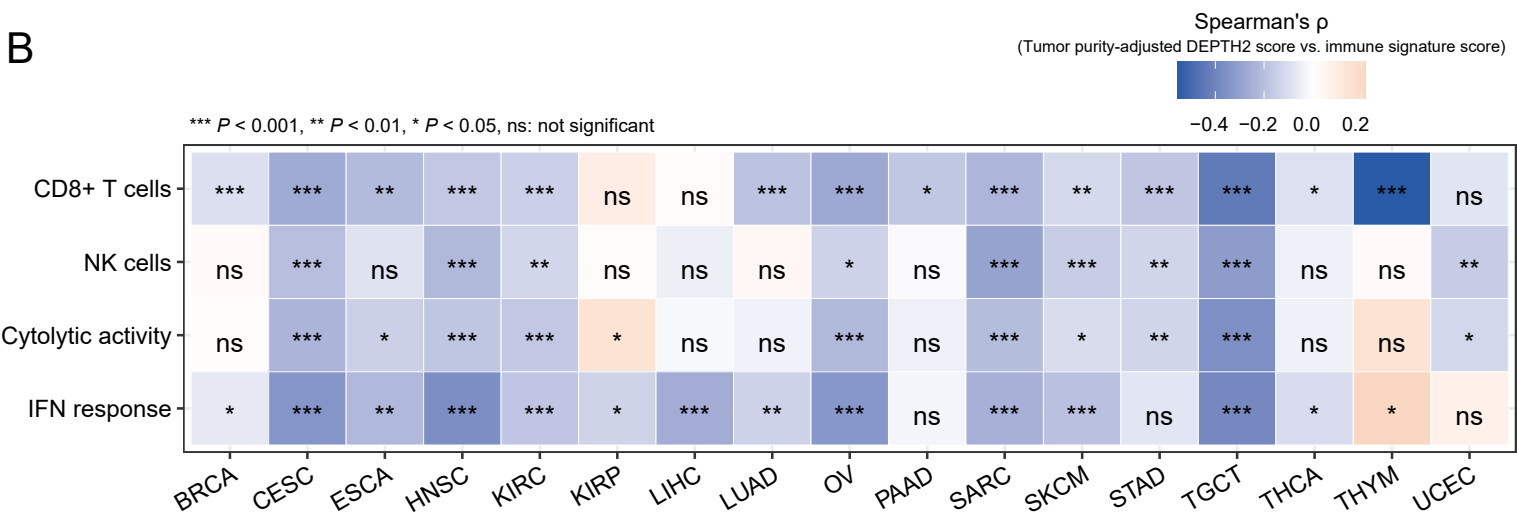

C

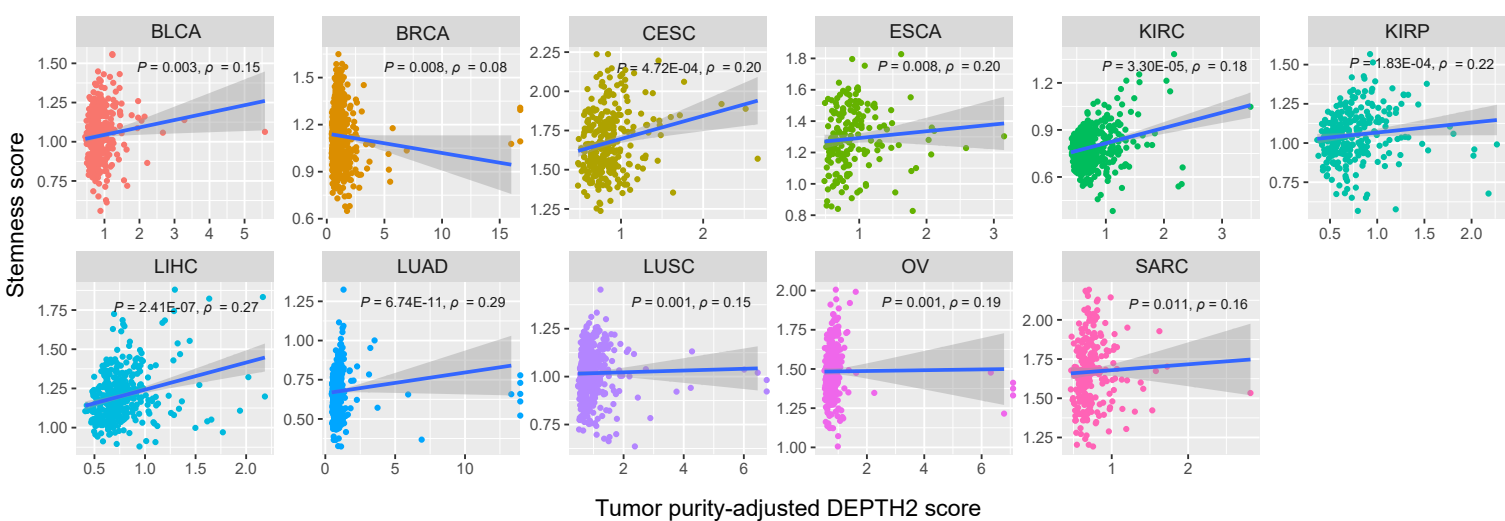

D

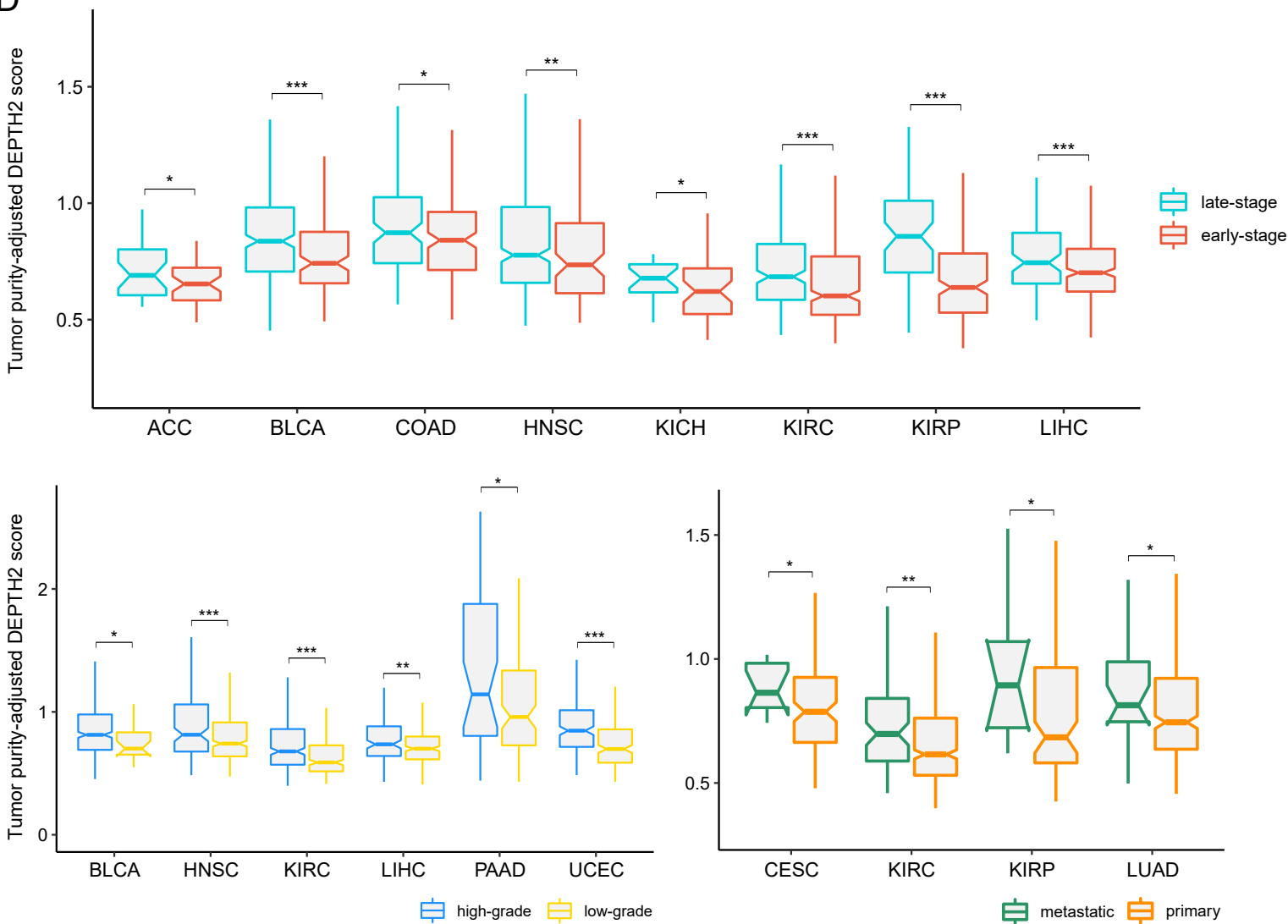

E

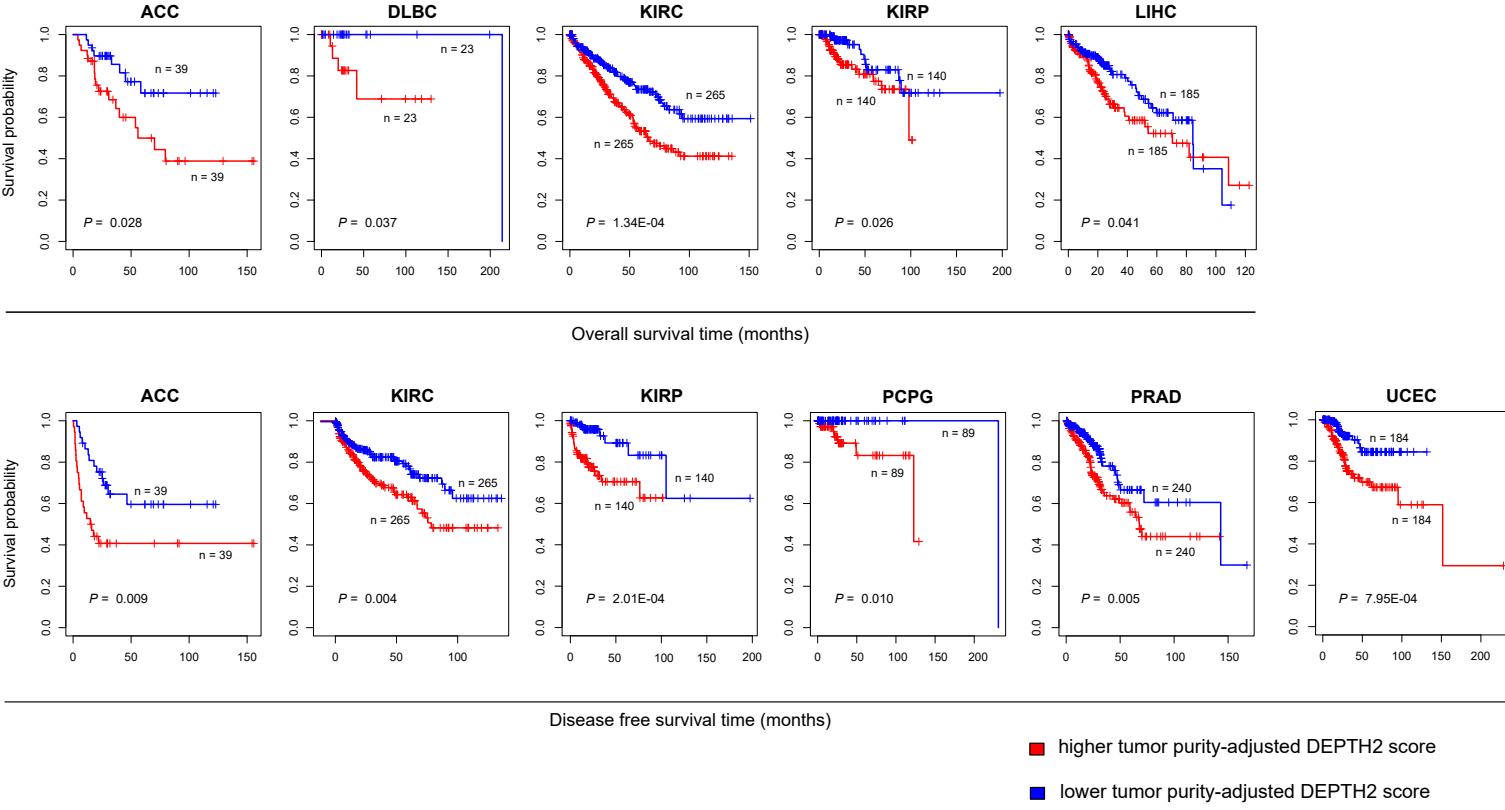

Supplement: Supplementary file 3 — Additional file 3: Figure S2. Associations of tumor purity-adjusted DEPTH2 ITH with genomic instability, antitumor immune signatures, tumor progressive phenotypes, and survival prognosis in cancer. (A) Significant positive correlations between tumor purity-adjusted DEPTH2 scores and genomic instability features (TMB, CNA, and HRD) in diverse cancers. (B) Significant negative correlations between tumor purity-adjusted DEPTH2 scores and antitumor immune signatures (CD8+ T cells, NK cells, immune cytolytic activity, and IFN response) in diverse cancers. (C) Significant positive correlations between tumor purity-adjusted DEPTH2 scores and tumor stemness scores in 11 cancer types. (D) DEPTH2 scores are significantly higher in advanced versus non-advanced [late-stage (stage III–IV) versus early-stage (stage I–II), high-grade (G3–4) versus low-grade (G1–2), and metastatic versus primary] tumors in diverse cancers (one-tailed Mann–Whitney U test, P < 0.05). (E) Kaplan–Meier curves showing that the tumors with higher tumor purity-adjusted DEPTH2 scores (> median) have more inferior overall survival and disease-specific survival than the tumors with lower tumor purity-adjusted DEPTH2 scores (< median) in 5 and 6 individual cancer types, respectively. The log-rank test P-values are shown. [file 12967_2022_3355_MOESM3_ESM.pdf]
